# Facial responsiveness of psychopaths to the emotional expressions of others

**DOI:** 10.1371/journal.pone.0190714

**Published:** 2018-01-11

**Authors:** Janina Künecke, Andreas Mokros, Sally Olderbak, Oliver Wilhelm

**Affiliations:** 1 Department of Psychology, Humboldt-Universität zu Berlin, Berlin, Germany; 2 Psychologische Hochschule, Berlin, Germany; 3 University Hospital of Psychiatry Zurich, Department for Forensic Psychiatry, Zurich, Switzerland; 4 Department of Psychology, Ulm University, Ulm, Germany; Universitatsklinikum Tubingen, GERMANY

## Abstract

Psychopathic individuals show selfish, manipulative, and antisocial behavior in addition to emotional detachment and reduced empathy. Their empathic deficits are thought to be associated with a reduced responsiveness to emotional stimuli. Immediate facial muscle responses to the emotional expressions of others reflect the expressive part of emotional responsiveness and are positively related to trait empathy. Empirical evidence for reduced facial muscle responses in adult psychopathic individuals to the emotional expressions of others is rare. In the present study, 261 male criminal offenders and non-offenders categorized dynamically presented facial emotion expressions (angry, happy, sad, and neutral) during facial electromyography recording of their corrugator muscle activity. We replicated a measurement model of facial muscle activity, which controls for general facial responsiveness to face stimuli, and modeled three correlated emotion-specific factors (i.e., anger, happiness, and sadness) representing emotion specific activity. In a multi-group confirmatory factor analysis, we compared the means of the anger, happiness, and sadness latent factors between three groups: 1) non-offenders, 2) low, and 3) high psychopathic offenders. There were no significant mean differences between groups. Our results challenge current theories that focus on deficits in emotional responsiveness as leading to the development of psychopathy and encourage further theoretical development on deviant emotional processes in psychopathic individuals.

## Introduction

Individuals with high psychopathic trait levels (referred to throughout this manuscript as psychopaths) come to about 1% of the general population [1, 2]. Psychopaths are responsible for a disproportionate number of crimes. According to criminological cohort studies, persistent offenders [many of whom are psychopaths] are responsible for more than 50% of the officially recorded offenses [3]. Furthermore, psychopathy is considered a primary risk factor for violent, serious, and repeat offending [4]. Phenomenologically, psychopathy is characterized by emotional detachment [5, 6]. Psychopaths seem to have shallow emotional experiences, they appear indifferent towards the feelings of others, and remorseless with respect to their harmful actions against others [7].

### Facial muscle responses as emotion simulation

In order to feel empathic concern for someone else, the internal simulation of the perceived emotional state is thought to be a crucial component [8, 9, 10]. Simulation processes involve the partial activation of affective, sensory, and motor systems associated with the perceived emotion [11]. Immediate facial muscle responses matching perceived emotional expressions [12, 13], so-called facial mimicry [14], are the expressive motor component of a simulated emotion [15]. Facial mimicry occurs in minimally affiliative contexts [16] and has been shown to be positively related with trait empathy [17, 18, 19]. Interestingly, the amount of facial mimicry can be manipulated experimentally. Inducing a competitive context in healthy adults led to reduced facial mimicry [20, 21], presumably because a competitive context lowered the participants’ empathic concern for their competitors. While psychopathy is associated with lower levels of self-reported empathy [22] and empathic accuracy [23], there is limited evidence regarding facial mimicry in psychopaths, and whether immediate facial muscle response to the emotional expressions of others is also reduced.

### Psychopathy and facial muscle responses to emotional stimuli

Hagenmuller, Rössler, Endrass, Rossegger, and Haker [24] found that psychopathic offenders were less likely than controls to mimic yawns and laughs. Adolescents with disruptive behavior disorder, which is characterized by hostile, aggressive, and deviant behavior, showed less congruent facial activity than controls in response to empathy-inducing film clips with sad and happy, but not angry, protagonists [25]. Younger boys with disruptive behavior disorder, on the other hand, showed less facial mimicry only to angry, but not happy, expressions [26].

Studies investigating facial muscle responses to emotional non-face stimuli in psychopaths also yielded mixed results regarding emotion-specific reductions in facial responsiveness. Herpertz et al. [27] did not find the expected valence effect in response to positive, neutral, and negative IAPS pictures in psychopaths. In comparison to controls, their corrugator activity in response to unpleasant pictures was not enhanced. Fanti, Panayiotou, Lombardo, and Kyranides [28] found a reduced facial response to violent, but not comedy, video-clips in individuals scoring high on callous-unemotional traits, which include symptoms akin to psychopathy, such as shallow affect or a lack of empathy and remorse [29]. Overall, the published empirical evidence does not yield credible conclusions regarding facial responsiveness in adult psychopathic individuals to the emotional facial expressions of others. This is something we address in the current study.

### Psychopathy and emotion recognition

Theoretically, the simulation of perceived emotional expressions not only supports empathic concern, but also facilitates access to the particular emotional concept, especially, when the emotion recognition is not trivial, such as in studies with increasing task difficulty affording individual differences in performance [13, 30]. Thus, an examination of emotion perception abilities in psychopaths may inform expectations as to facial muscle responses to the perception of emotion expressed by others. The findings of individual studies on emotion perception are heterogeneous [31]. Hence, meta-analyses and systematic reviews will be considered in depth.

In their meta-analysis on emotion recognition abilities in antisocial populations, Marsh and Blair [32] found poorer emotion recognition performance by antisocial individuals for the recognition of fear and sadness, with effects largest for fear. They found no differences in performance when specifically comparing psychopathic and non-psychopathic antisocial individuals. Wilson, Juodis, and Porter’s [33] meta-analysis on emotion perception deficits in psychopaths found weak deficits in the perception of fear, happiness, and sadness expressed in the face. When focusing on tasks with a verbal response style, as is the case in the current study, emotion recognition deficits in the perception of fear, sadness, and anger increased. Finally, the meta-analysis by Dawel, O’Kearney, McKone, and Palermo [34] identified significant deficts in the ability to perceive emotion expressed through the face in general, with specific deficits in the ability to perceive fear, happiness, sadness, and surprise.

In their review on emotion processing and psychopathy, Brook and colleagues [31] found no clear pattern for an emotion-specific or emotion-general deficit in emotion perception abilities that could be traced to task characteristics. Additionally, there are several studies that did not yield support for any emotion perception deficits in psychopaths [35, 36, 37], with some even finding better overall performance [38].

In sum, behavioral evidence for emotion recognition deficits in psychopaths is not unequivocal. The contrasting findings could, however, be due to methodological limitations. Most of the individual studies just reviewed, as well as those included in the meta-analyses, relied on comparatively small samples, utilized only a single measure of emotion perception, often with poor internal consistency [34], and frequently involved data with distributional problems, such as average performance approaching the ceiling (e.g. [39]). In the present study, we aimed to prevent the outlined methodological shortcomings by using a large sample size and a state-of-the art statistical approach.

### The amygdala-deficit hypothesis

According to Blair’s integrated emotion systems (IES) theory [5, 22, 40], psychopathic individuals suffer from an amygdala dysfunction which causes impaired stimulus-reinforcement learning surrounding punishment and reward related stimuli, leading to reductions in related autonomic, attentional, and behavioral responsiveness. IES theory proposes that aversive or appetitive stimulus-reinforcement learning is initiated by ‘care-based’ emotional expressions (e.g., fear, sadness), which signal that the other person is in distress, and happy expressions, which serve as reinforcers within social interactions. As psychopaths do not develop these associations (e.g., relating perceived sadness with their harmful behavior towards the expresser), however, aggressive behavior becomes an unencumbered means to achieve one’s goals. Indeed, lower amygdala volume and reduced amygdala activity in response to emotional stimuli in psychopaths supports this view [40]. Moreover, Seara‐Cardoso and Viding [41] present support for reduced activity within brain areas typically associated with the processing of affect in psychopaths in response to the perception of fearful, sad, and happy expressions. These reductions in brain activity apply to the amygdala and emotion simulation areas [37], like the inferior prefrontal gyrus and superior temporal sulcus as well as visual cortical areas associated with feedback modulation by the amygdala [42]. Thus, the IES model explains emotion-specific recognition deficits of fearful, sad [32], and happy expressions [34], emotions preferentially processed in the amygdala [43], although results on the emotion-specificity of the amygdala are mixed (e.g., lesion study by Adolphs & Tranel, [44]).

### The present study

In the present study, we investigated emotion-specific processing deficits in psychopaths by examining their facial muscle responses to the perception of facial expressions of emotion. We recorded activity of the *m*. *corrugator supercilii*, which pulls down the eyebrows in a frowning expression, while participants conducted an emotion categorization task with dynamically presented angry, happy, and sad facial expressions.

While theoretical models about the etiology of psychopathy routinely identify deficits in the processing of fear [40, 45, 46], corrugator effects in response to fearful expressions [47, 48] or during fearful mental imagery [49] are not supported within the facial electromyography (EMG) literature consistently. Importantly, in a previous study, using a very similar experimental design and stimuli, there was no significant difference in corrugator activity between the perception of fearful and neutral facial expressions [30]. Furthermore, no other muscle has been identified in the literature whose activity is consistently associated with the perception of fear. Thus, we did not examine facial muscle responses to the perception of fear.

Similarly, we focused on the activity of the corrugator muscle, because it is a reliable index of facial mimicry for happy, angry, and sad expressions [50], in contrast to the zygomaticus, the muscle pulling up the cheeks in smiling. Concerning the zygomaticus, Künecke et al. [30], for example, found no significant differences in activity when participants viewed faces expressing different emotions. The corrugator has a high valence sensitivity [51], which might be due to lower voluntary control in comparison to the zygomaticus muscle [52] and dense connection to the amygdala [53]. Thus, given these arguments, we restricted our measurement to corrugator responses to the presentation of angry, happy, sad, and neutral expressions.

We aimed to replicate a measurement model of facial muscle responses to emotional expressions [30] that controls for general facial responses to face stimuli and models emotion-specific facial response factors, which were significantly related to emotion perception ability. In confirmatory factor analysis (CFA; [54]) latent variables (factors) are predicted by several manifest variables (indicators), thought to measure the same construct. The resulting factors represent the underlying construct predicting responses to that measure, but without the measurement error. In the present study, we compare the latent means of the emotion-specific response factors in high and low psychopathy offenders, and non-psychopathic non-offenders. For ease of presentation, we report group differences. For the sake of completeness, structural equation models with a continuous measure of psychopathy are provided in an appendix (see supporting information S1 Fig). Likewise, group differences in the emotion-specific factor correlations are presented in the supporting material (S1 Tables).

First, we report average accuracy scores for the emotion categorization task, in addition to average reaction time, in order to ensure generally appropriate task performance. Then, with multiple-group confirmatory factor analysis (MCFA; [55]), we checked for strong measurement invariance of the corrugator response model (i.e., equality across groups in factor structure, factor loadings, and indicator intercepts). Strong measurement invariance assures that the measurement model is appropriate to describe the relations between manifest and latent variables independent of group. This allows to compare the means of the latent factors. Based on the amygdala-deficit hypothesis we predicted lower emotion-congruent corrugator responses for the high psychopathy offender group in comparison to the low psychopathy offender and the non-offender groups. More specifically, we assumed that these deficits would be limited to responses toward happy and sad, but not angry expressions, as the former are considered to be processed by the amygdala in particular [43].

## Methods

### Ethics statement

The research plan was approved by the Department of Psychology of the Humboldt-Universität zu Berlin ethics committee as well as by the individual prison judicial review boards and regional authorities in charge of the forensic-psychiatric hospitals where the data collection took place. In addition, each participant was asked to provide separate written informed consent for taking part in the study, for granting access to his criminal record (for incarcerated participants only), for allowing gene analysis (collected through buccal swabs), EMG recordings, and the use of video recordings. The experimenter individually checked back if the participants understood the consent forms before signing. After completion of the study, participants received financial compensation for their participation.

### Participants

For the two offender groups, we recruited male inmates from eight German forensic-psychiatric hospitals and correctional facilities. Participants were recruited immediately prior to and during data collection as a specific institution, from 2013 to 2015. For the non-offender group, we recruited persons from the forensic-psychiatric hospital staff and by targeting a community sample in two German cities through online advertisements and by placing leaflets in locations that are frequented by individuals of lower socio-economic status (e.g., agencies that hire for temporary employment positions, or provide unemployment benefits). Specifically, we tried to match the community sample with the incarcerated participants in terms of their socioeconomic status and education level [56].

Exclusion criteria for all participants were a current diagnosis of psychosis, such as schizophrenia, and an IQ below 75. In total, 347 persons took part in the study. The data from 14 was excluded because they met our exclusion criteria and another 25 individuals had no or incomplete EMG data. Due to recording problems, another 42 participants showed insufficient EMG data quality, which led to an EMG-sample size of 266 individuals (179 inmates and 87 controls). For the final sample, the age of the participants ranged from 19 to 68 years (*M* = 35.85, *SD* = 11.02) and 87% of the participants were German nationals. Educational background was heterogeneous: most of the sample had completed a vocational-track or intermediate-track school (61%; *Hauptschule* or *Realschule*), 27% had completed an academic-track degree (*Gymnasium*) or higher, and 10% did not graduate from a school that could be deemed equivalent to high-school level. The offences committed by the inmates varied from theft, fraud, robbery, and drug-related crimes to rape, manslaughter, and multiple homicide.

Psychopathy was assessed with the *Psychopathy Checklist*: *Screening Version* (PCL: SV; [7]). In the full sample, PCL:SV scores ranged from 0 to 24 (*M* = 9.70, *SD* = 6.70). In German-speaking countries, based on data from a meta-analysis with seven studies and a total-*N* of 1,124 [57], the PCL:SV cutoff for the diagnosis of psychopathy has been recommended at 17 points. Incorporating one standard error of measurement [*SE* = 2; (7)], we utilized a cutoff score of 15 in order to minimize the false-negative rate. Psychopathy, at least as measured with the PCL: SV and the PCL-R [58], is a dimensional construct. The PCL: SV cut-score used in the present study (i.e., 15 out of 24 points = 5/8) coincides with the margin at which total scores on the PCL-R would be considered as “high” ([57] p31; i.e., 25/40 = 5/8). The offender groups are henceforth labeled as *low psychopathy* and *high psychopathy*. In the non-offender group, the PCL:SV scores ranged from 0 to 13 (*M* = 2.29, *SD* = 2.83, *n* = 86), from 2 to 14 in the low psychopathy group (*M* = 9.59, *SD* = 3.20, *n* = 97), and from 15 to 24 in the high psychopathy group (*M* = 17.49, *SD* = 2.36, *n* = 83). The low and high psychopathy groups comprised inmates exclusively with one exception. One of the non-offender participants had a PCL:SV score of 18 and was thus included in the high psychopathy group. One participant gave informed consent to participate in the study, but refused to grant access to his prison files. In this case, the PCL:SV was scored from interview information only.

### Design and procedure

The study consisted of two testing sessions. During the first session (lasting for approximately 2.5 hours with four breaks of about 5 minutes each) participants completed a computerized battery of psychological tests, including the emotion categorization task during which corrugator activity was assessed with EMG. The task battery included 14 cognitive tasks and three self-report questionnaires, which were programmed and administered with Inquisit 4.0 [59] and presented on a 24-inch monitor. Other data collected through this study, but not used in the present paper, are published elsewhere [60]. The emotion categorization task presented in the present study was the third task administered, but the first emotion-related and EMG task. The EMG recording started after the first break. The second testing session, completed one or two days after the first session, depending on the participant’s schedule, lasted for one hour. During the second session, participants were interviewed for the PCL:SV rating [7] and the antisocial personality disorder items from the Structured Clinical Interview for DSM-IV, section II (SKID-II; [61]). Then, those who consented gave their saliva sample for later genetic analysis. In the present study, we present the results of the EMG and PCL:SV data. Further test, video, and gene results will be reported elsewhere. All PCL:SV interviews were administered and scored by interviewers who had undergone an official PCL:SV training by a Darkstone-accredited PCL-R/SV instructor. For the offender samples, the participants’ criminal files were also reviewed in order to prepare for the PCL:SV interview and determine the subsequent ratings.

### Measures

#### Psychopathy Checklist: Screening Version (PCL:SV)

Psychopathy was assessed with the PCL:SV **[7]**), an abridged 12-item version to the longer Psychopathy Checklist-Revised [62], which is recommended for use outside of forensic settings [63]. According to the manual, “[because] the PCL:SV can be completed in the absence of criminal record information, it is more appropriate than the PCL-R for use outside of forensic settings” ([7] p2), for instance in studies involving individuals sampled from the community at large. In general, scoring is based on both available file information and the information retrieved in a semi-structured interview. We used an interview guide covering the following areas: “presenting problem/current legal status, educational history and goals, vocational history and goals, medical and psychiatric history, family background, marital history, juvenile conduct problems, adult antisocial behavior (including substance abuse)” ([7] p18). Items are coded on a three-point rating scale (0 *= no*, 1 *= maybe*, and 2 *= yes*). The PCL:SV items are summed to create a total scale score representing that person’s psychopathic trait level. Up to two items may be omitted while still allowing to obtain a total score for that participant. In the case of missing data, the final score was prorated according to the instructions from the manual.

According to the PCL:SV manual [7], the inter-rater agreement of the PCL:SV total score is high (median intra-class correlation coefficient [ICC] = .82). These estimates of inter-rater agreement were obtained by comparing the assessments of single raters pooled across different samples (inmates, civil psychiatric patients, and students). For the current study, the ratings of 13 cases were compared with those made by two experienced forensic psychologists who had undergone an accredited 2-day PCL-R/SV training workshop. The ICC(1,1) coefficient (one-way random, single measure, absolute agreement) for the PCL:SV total score was .80, with a 95% CI of [.48, .93]. According to Landis and Koch ([64] p165), ICC values of .41 or above can be regarded as "Moderate", those of .61 or above can be considered "Substantial", and ICC values of .81 or above can be considered "Almost Perfect".

#### Emotion categorization

During the EMG measurement, participants were asked to categorize dynamic angry, happy, neutral, and sad facial expressions. The expressions were morphed colored videos (30 frames per second) 8 x 12 cm in size, presented in the center of a light grey background. The stimuli consisted of 38 target persons (19 females) from the Radboud database [65] and were morphed with a morphing software [66]. These stimuli had been used successfully in a prior emotion recognition task also completed during facial EMG recording [30]. Every target was shown twice for each of the four emotions. In total, participants were asked to categorize 38 (targets) x 4 (emotions) x 2 (repetitions) = 304 expressions, which were presented in a fixed random order. Each trial began with a fixation cross for 700ms. Then, within 200ms, the neutral expression turned into the full emotional expression. In the neutral condition, we morphed neutral photos to create a video of a person blinking. The full expression was then shown for another 400ms, followed by a blank screen for 500ms, after which the next trial began. Participants were asked to indicate, to the best of their ability as and quickly as possible, the emotion expressed by the target by pressing the corresponding key on the keyboard. Participants were instructed to hold their index and middle fingers of their left and right hands on the corresponding keys (“y”,”x”,”n”, and “m”) during the whole experiment on a standard QWERTZ keyboard. The stimulus-response order was counterbalanced across participants. Before the actual experiment began, there were two practice trials to make sure the participants understood the instructions. After half of the trials, there was a short break of self-paced duration.

### EMG recording

We recorded facial muscle activity with 3 Ag/AgCl electrodes that were 4 mm in diameter. According to EMG guidelines, two shielded electrodes measured the activity of the corrugator on the left side of the face [67]. In addition, one un-shielded electrode was placed on the middle of the forehead to serve as a grounding electrode. Participants’ skin was first peeled and cleaned with alcohol before the electrodes, filled with conductive gel, were attached. Impedances were kept below 15 kΩ. Data were amplified and bandpass-filtered online (10-500Hz) with an EMG100C module and sampled at 1000 Hz by the digital converter system MP150 (Biopac Systems Inc., USA). An additional Isolated Digital Interface (STP100C) recorded stimulus presentation times.

### EMG preprocessing

Offline continuous raw data were notch-filtered at 50 Hz and high-pass filtered at 20 Hz, to reduce motion artifacts [68], in Acknowledge 4.1 software (Biopac Systems Inc., USA) and then exported to R Environment for Statistical Computing [69] for further processing. We conducted a full-wave rectification and moving average with a time-window of 50 ms for quantification and smoothing of the continuous data. Data were segmented in 1500 ms time intervals with a pre-stimulus baseline of 500 ms. After baseline correction, we performed an automatic artifact detection. All trials with an incensement of more than 3.5 *SD* of the individual’s overall mean activity within 50 ms time intervals were excluded [30]. Additionally, we discarded trials with reaction times below 300 ms and above 6000 ms. This led to an average exclusion of 17.5% of the trials per participants. Then, the data were *z*-standardized within individuals to overcome large individual differences in general EMG activity [70]. Finally, we excluded all incorrect trials (on average 6.1% of the trials per participant).

**Fig 1:**
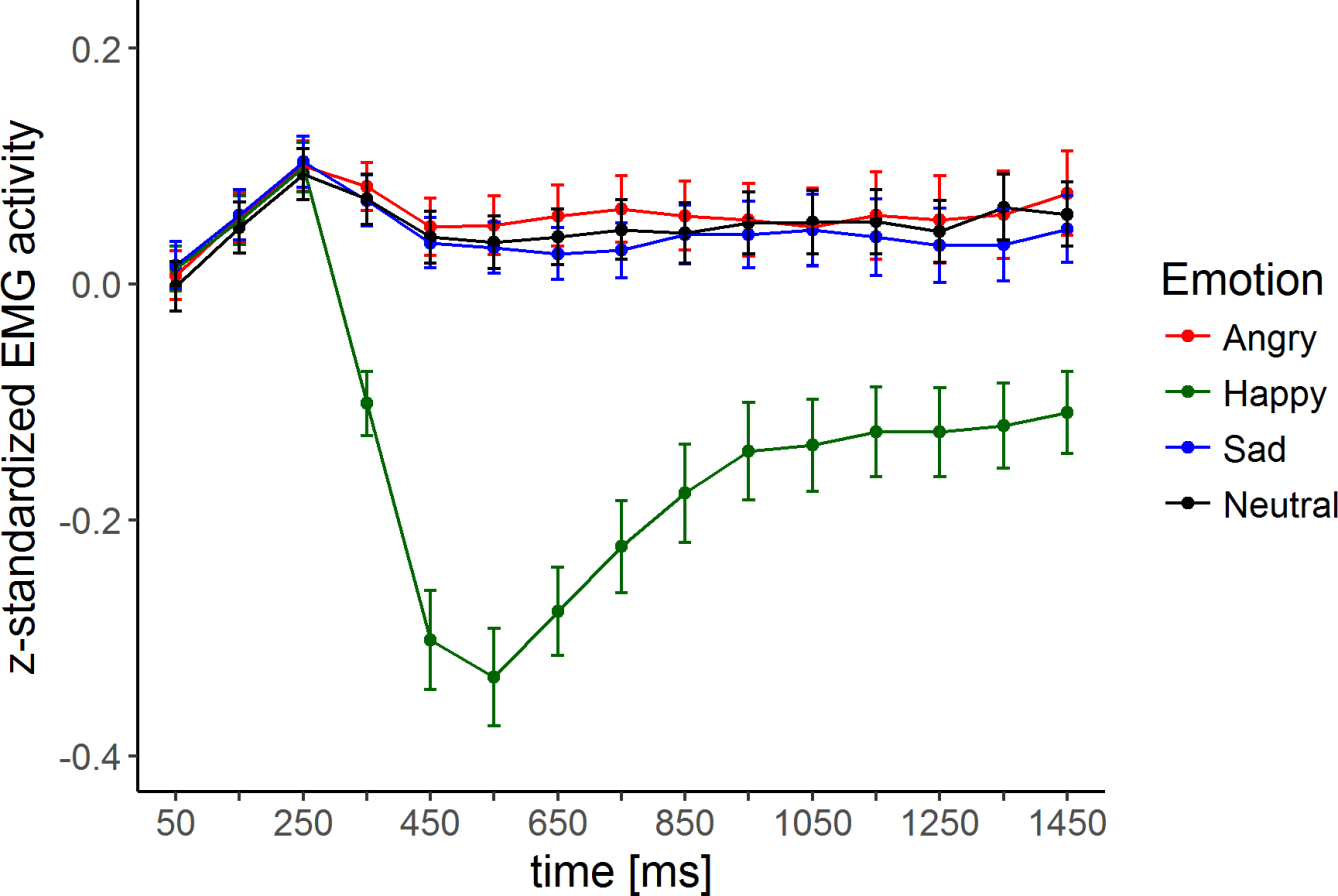
Time course of corrugator mean activity after stimulus onset seperately for each emotion category. Errors bars depicts standard errors.

For the indicators of the measurement model, we averaged mean activity in the 300 to 800 ms time window for one-third of the trials within each emotion category. This resulted in three indicators per emotion category for one latent factor: an1-an3 for the anger factor (ANG), ha1-ha3 for the happiness factor (HAP), sa1-sa3 for the sadness factor (SAD), with an1-an3, ha1-ha3, sa1-sa3, and ne1-ne3 as indicators of the general face factor (FACE). This time window has been used in a previous study [30] and seems to adequately reflect the emotion effect in this study too (see Fig 1 for the time course of the EMG responses for each emotion category or see [71] for a comparable design and corrugator effect). For the measurement models, ha1-ha3 were reversed so that high values for all indicators indicate more congruent facial muscle responses. Reliable averaged EMG responses required at least 5 trials per indicator per participant. Five participants failed to meet this criterion and hence were excluded from later analyses. Thus, the final sample size was *n* = 261 (86 controls, 95 low psychopathy and 80 high psychopathy individuals).

### Statistical analyses

All statistical analyses were performed in *R* [69]. We tested mean differences in accuracy and reaction time scores for the emotion categorization task and corrugator activity, as assessed with the EMG, in a repeated-measures ANOVA with group membership as the between-subject factor and emotion as the within-subject factor. The dependent variable was corrugator activity during the pre-defined time window of 300 to 800 ms after stimulus onset [30]. We estimated the repeated-measures ANOVAs with the *ezAnova* function of the *ez* package. Type I error rates in post-hoc comparisons were Bonferroni-corrected, at a familywise error rate of .05.

**Fig 2:**
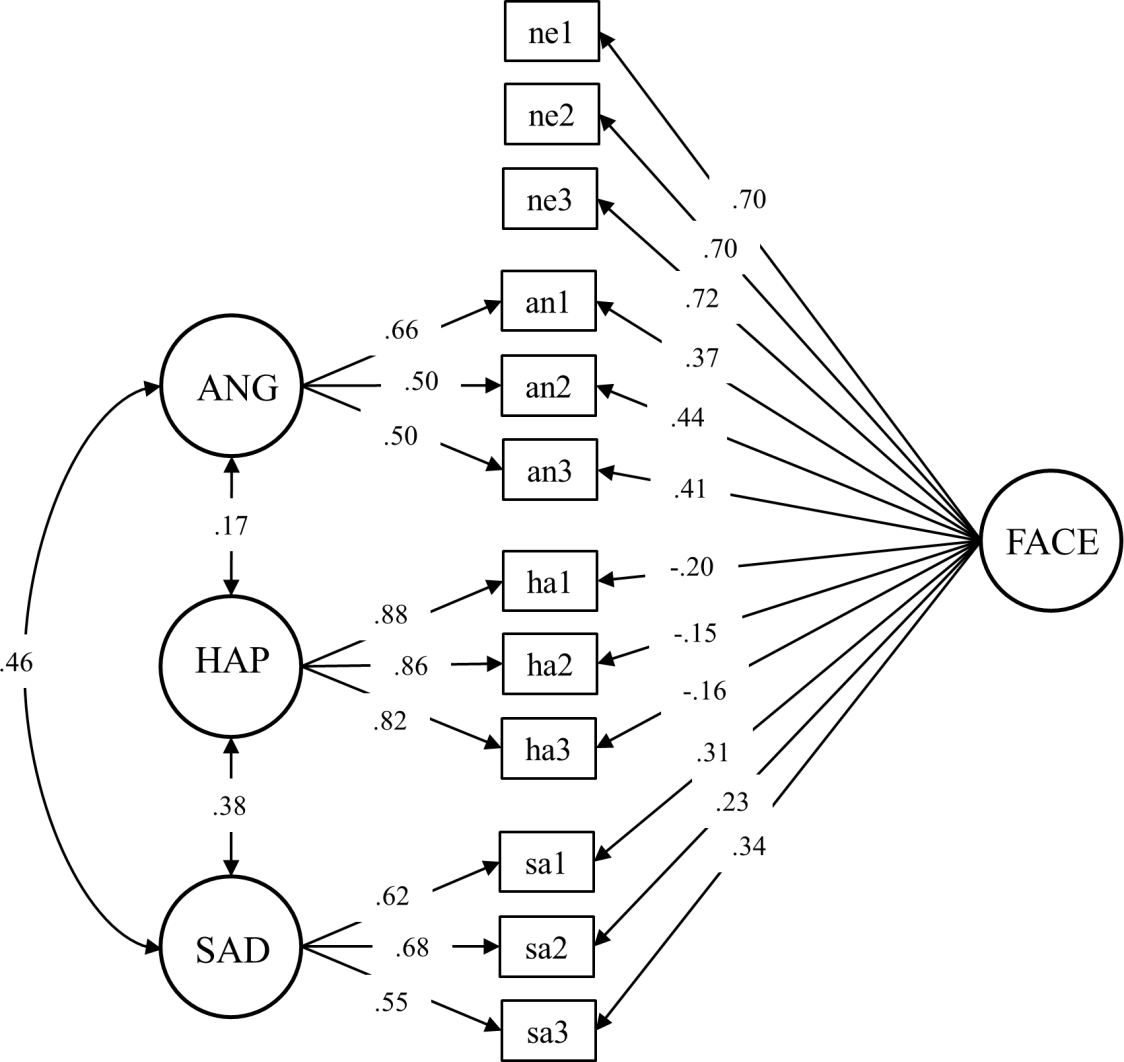
Measurement model of facial muscle responses for the whole sample.

We applied CFA [54] to model individual differences in corrugator activity. CFA models were estimated with the *sem* function of the *lavaan* package [72] using the maximum likelihood (ML) estimator. To replicate the corrugator response model by Künecke et al. [30], we modeled one general face response factor loading on all indicators and three correlated emotion-specific factors for corrugator responses loading on the angry, happy, and sad expression indicators, respectively. The measurement model is depicted in Fig 2. Squares represent the indicators (i.e., manifest variables; what we actually measured) and circles represent the latent factors. The latent factors predict the manifest variables, indicated by direct paths, e.g. the arrow from FACE to ne1 in Fig 2. These regressions weights are called factor loadings and represent the strength of the relationship between the latent and the manifest variables. Two-headed arrows represent correlations among the latent factors, e.g. between the emotion-specific factors ANG and SAD. Latent factors were scaled with effects coding [73] with the sum of all indicators within one factor fixed to one and the sum of the intercepts within one factor fixed to zero. This allows factor loadings, factor variances, and factor means to be freely estimated and for the factor means to be estimated in the unit of the manifest indicators. With the iterative ML method model parameters are estimated in way that maximizes the similarity between the covariance matrix implied by the assumed model and the observed covariance matrix. Higher similarity indicates a better model fit, for which we report the following indices: Acceptable model fit would be considered present if the Comparative Fit Index (CFI) value was above .95 and the Root Mean Square Error of Approximation (RMSEA) as well as the Standardized Root Mean Square Residual (SRMR) values were below .08 [74].

We tested measurement invariance of the measurement model across our three groups using multiple-group confirmatory factor analysis (MCFA). First, the assumed measurement model was estimated for all three groups separately to ensure factorial validity [55]. Measurement invariance was then tested by comparing nested models with an increasing number of model constraints (i.e., same factor structure and factor-indicator relationships followed by equating loadings followed by equating intercepts) with χ^2^-difference tests [75]. Additionally, we report ΔCFI for model comparisons. Non-significant χ^2^-difference tests and ΔCFIs < .002 [76] indicate that invariance constraints hold across groups. Strong factorial invariance is given when factor-indicator relationships (configural invariance), factor loadings (metric invariance), and indicator intercepts (scalar invariance) are equal across groups [77, 78], which is sufficient to interpret latent means across groups [79]). Finally, we compared models with equality constraints on the factor means to test our hypothesis.

## Results

### Accuracy, reaction time, and corrugator activity

Table 1 depicts the descriptive statistics for performance accuracy, reaction time, and mean EMG activity during the time-window of 300 to 800 ms for each emotion category per group. There was a significant effect of emotion on performance accuracy, *F*_(3,774)_ = 92.41, *p* < .001, η^2^ = .17. Pairwise comparisons showed that happy expressions were better recognized than the other expressions. Identification of sad expressions was lower than that for the other expressions, while anger and neutral expressions were recognized equally well. There was no significant effect of group and no significant interaction between emotion and group.

**Table 1:** Descriptive statistics, M (SD), for accuracy, reaction time and mean EMG activity in the 300–800 ms time-window for all emotion categories per group; NO = _non offender group, LP = low psychopathy group, HP = high psychopathy group.

Emotion	Accuracy			Reaction Time [ms]			Corrugator activity [z-standardized mean activity]		
	NO	LP	HP	NO	LP	HP	NO	LP	HP
Anger	.91 (.08)	.91 (.08)	.90 (.09)	1624 (395)	1818 (491)	1847 (532)	.07 (.15)	.05 (.18)	.07 (.17)
Happiness	.98 (.02)	.99 (.02)	.99 (.01)	1215 (354)	1325 (385)	1366 (368)	-.26 (.23)	-.27 (.28)	-.20 (.26)
Neutral	.94 (.08)	.92 (.13)	.91 (.12)	1441 (399)	1610 (441)	1824 (657)	.03 (.13)	.03 (.15)	.06 (.18)
Sadness	.88 (.09)	.87 (.13)	.88 (.09)	1809 (468)	1949 (526)	1991 (541)	.06 (.18)	.05 (.15)	.04 (.18)

The significant effect of emotion on reaction time, *F*_(3,774)_ = 240.50, *p* < .001, η^2^ = .19, mirrored this pattern. Happy expressions were recognized the fastest while sad expressions were recognized the slowest. There was also a significant effect of group, *F*_(2,258)_ = 8.75, *p* < .001, η^2^ = .04, with the non-offender group being faster at recognizing facial expressions when compared with the low and high psychopathy groups. The significant emotion × group interaction, *F*_(6,774)_ = 3.72, *p* < .001, η^2^ = .01, indicated that the high psychopathy group was slower at recognizing neutral expressions in comparison to the low psychopathy group, but their reaction times did not differ for the other emotion categories.

Facial EMG activity during the 300 to800 ms time window also showed a significant effect of emotion, *F*_(3,774)_ = 179.74, *p* < .001, η^2^ = .31. There was significantly reduced corrugator activity while viewing happy facial expressions, in comparison to all other conditions, which supports our decision to reverse the ha1- ha3 indicators for the later measurement model, so that high values for all indicators means more congruent facial muscle responses. There was no difference in the corrugator activity while viewing angry, sad, and neutral facial expressions and there was no significant group or emotion × group interaction effect.

### Confirmatory factor analysis of corrugator activity

The corrugator measurement model for the whole sample is depicted in Fig 2. The model contains one general face response factor (FACE), which controls for emotion-unspecific variance in all indicators, and three correlated emotion-specific factors representing emotion specific facial responses to angry (ANG), happy (HAP), and sad (SAD) facial expressions. The model fit was good, χ^2^_(42)_ = 60.00, *p* = .03, CFI = .984, RMSEA = .041, SRMR = .029. All factor loadings were significant, except for ha2 and ha3 on FACE (*p* = .07/.06). The factor correlations were also significant.

### Multiple-group confirmatory factor analysis of corrugator activity

Before we could test our hypothesis, we first needed to establish that the corrugator measurement model was invariant between the three groups. Model fit was still acceptable when estimated for the three groups separately: non-offenders, χ^2^_(42)_ = 46.43, *p* = .03, CFI = .985, RMSEA = .035, SRMR = .060; low psychopathy, χ^2^_(42)_ = 63.07 *p* = .02, CFI = .960, RMSEA = .071, SRMR = .045; and high psychopathy, χ^2^_(42)_ = 41.82, *p* = .48, CFI = 1.000, RMSEA < .001, SRMR = .051. Next, we modeled a MCFA with configural invariance and successively applied constraints on the factor loadings (metric invariance) and indicator intercepts (scalar invariance). We found invariance at each step (see Table 2 for the fit indices and test statistics for the invariance comparisons across the three groups). Thus, the model displayed in Fig 2 fit the three groups equally well. Consequently, further comparisons between the groups on factors means were admissible. We calculated the construct reliability of the corrugator response factors with weighted omega ω_W_, which represents the shared variance of all indicators of a latent factor [80]. Reliabilities of all latent factors were acceptable: ω_W_ FACE = .80, ω_W_ ANG = .59, ω_W_ HAP = .89, and ω_W_ SAD = .66.

**Table 2:** Results for measurement invariance testing across groups.

Model	χ^2^	df	CFI	RMSEA	SRMR	Compared to model	Δχ^2^	Δdf	*p*-value	Δ CFI
1) Configural invariance	150.32	126	.979	.047	.052					
2) Metric invariance	186.29	160	.978	.043	.084	1	35.97	34	.38	.001
3) Scalar invariance	193.72	176	.985	.034	.084	2	7.43	16	.96	-.007

Next, in order to find out whether there were significant differences between the groups on the FACE and the emotion-specific corrugator response factors, we set all factor means to equality. There was no significant decrease in model fit (Δχ^2^_(8)_ = 6.50, *p* = .59, ΔCFI = -.001) indicating that there was no significant difference in either general or emotion-specific corrugator activity between the three groups, thus disconfirming our hypothesis. Table 3 shows the freely estimated latent means and Cohen’s *d* effect size before the constraints were applied. Note that all scores are positive, since happy scores were reversed before they were included in the CFA analysis. Higher values indicate higher emotion congruent facial responsivity. Complementary analyses using a continuous latent variable of psychopathy mirrored the results of the group comparisons (please see supporting information S1 Fig).

**Table 3:** *Latent factor means in the model with scalar measurement invariance;* Unstandardized estimates (M) [individually z-standardized mean activity], standard errors (SE), standardized estimates (μ), and Cohen’s d effect size estimates for group comparisons.

Latent Factor	non-offender (NO)			low psychopathy (LP)			high psychopathy (HP)			Cohen’s *d*		
	*M*	*SE*	μ	*M*	*SE*	μ	*M*	*SE*	μ	NO vs. LP	NO vs. HP	LP vs. HP
FACE	.013	.006	.277*	.012	.007	.213	.028	.009	.402*	.02	-.22	-.21
ANG	.049	.015	.487*	.029	.017	.215	.034	.018	.317*	.13	.09	-.03
HAP	.270	.026	1.218*	.276	.028	1.075*	.217	.030	.887*	-.02	.21	.22
SAD	.042	.014	.412*	.034	.014	.334*	.006	.021	.035	.06	.22	.17

* indicate p-values < .05

## Discussion

In the present study, we investigated corrugator activity in response to facial expressions of emotion in non-offenders and in criminal offenders who had varying levels of psychopathy. First, we aimed to replicate a measurement model of immediate facial responses in corrugator activity to emotional expressions [30]. Then, we tested measurement invariance across groups, including comparisons of latent means between the three groups in order to test differences in the size of facial responses to emotional expressions. The model from Künecke et al. [30] was structurally replicated and scalar invariance held across groups. This model has the core advantage of controlling for general facial responsiveness to facial emotional expressions, which then allows one to derive purer estimates of emotion-specific facial muscle responses. The means of the emotion-specific factors were then compared between the three groups.

### Intact emotional responsiveness in psychopaths

The amount of corrugator activity did not differ significantly between the groups, disconfirming our first hypothesis. This means that in our study emotion simulation on the facial muscle level, seen as a constitutive component of emotional concern for others [8, 9, 10], was not disrupted for psychopaths. Thus, emotional detachment, a core component of psychopathy, seems to be unrelated to immediate facial muscle responses toward angry, happy, or sad facial expressions.

Our lack of group differences contradicts Hagenmuller et al. [24], who found less emotional contagion for smiles and yawns by psychopaths, in comparison to healthy controls. However, the sample tested by Hagenmuller et al. was relatively small (12 psychopathic offenders and 10 controls) and mimicry was assessed via subjective video ratings made by one rater only, while we objectively measured highly sensitive EMG responses. Likewise, our results contradict the findings of de Wied et al. [25] who reported an EMG pattern of reduced facial responsiveness to sad and happy expressions in adolescents with disruptive behavior disorder. The stimulus material used in the current study, however, differed substantially from the material employed in the study by de Wied and colleagues [25]. In the de Wied et al. study [25] participants watched video clips intended to induce empathy for more than two minutes in duration. These video clips entailed narratives involving anger, happiness, or sadness in which the stimulus persons expressed intense facial and vocal responses. Thus, we suggest that de Wied et al. [25] likely measured deliberate empathic concern rather than immediate facial responsiveness. Eisenbarth, Alpers, and Kosson [81] found that lower facial muscle activity in response to positive and negative facial expressions and IAPS pictures were assocaited with the affective factor of psychopathy. In their experimental paradigm, however, participants were instructed to deliberately respond with frowning or smiling to the stimuli. In our study, facial muscle activity represents immediate, uncontrolled responses to emotional expressions.

The current results accord well with findings from a recent study using event-related potentials (ERPs; [82]). According to the study by Decety et al. [82] early automatic ERP components hardly associated with psychopathic trait levels whereas later (deliberate) components were. Similarly, the lack of empathy commonly ascribed to psychopaths may be due to an unwillingness to empathize rather than an inability to mirror the emotional states of others. On the other hand, the seemingly contradictory results reported by Herpertz et al. [27], namely muted facial expressions of psychopaths compared to controls in an EMG task, may have to do with stimulus content: While we used pictures of faces showing facial expressions, Herpertz et al. [27] used strong positive and negative images inducing high arousal levels (such as pictures of mutilated bodies or other violent imagery). Moreover, Herpertz et al. [27] used the maximum EMG response within a 500 to 3000 ms timeframe following stimulus onset as their variable of interest. In this regard, our experimental setup may be more akin to the early processing components described by Decety et al. [82], whereas the setup used by Herpertz et al. [27] may have reflected the late processing components.

It should be emphasized that our sample was at least six times larger than the samples tested by the aforementioned researchers. Consequently, the greater statistical power afforded by the larger sample would likely have identified even subtle differences if they had been present. Through MCFA we were able to account for measurement error, control for facial responses to face stimuli in general, and–importantly–to ensure measurement invariance across groups. Likewise, in a structural equations model involving psychopathy as a continuous trait, psychopathy was not related to any corrugator response factors (see supporting information S1 Fig). The latent variable approach is superior to mean comparisons of observed measures because the method of structural equation modeling controls for error variance. It should be noted, however, that the conclusions drawn from the latent variable analysis were in agreement with the results found at the level of the manifest variables in the current study.

### Limitations

Potentially, the small corrugator response effects were not sensitive enough to capture group differences in emotion-specific corrugator responses factors. The average emotion effects in the corrugator activity (see Fig 1) were present for happy expressions and absent for angry and sad expressions, contradicting Künecke et al. [30]. This might be due to differences between the two studies in their experimental setting and sample characteristics. In the present study, only angry, happy, sad, and neutral expressions were used, while Künecke et al. [30] additionally presented disgusted, fearful, and surprised expressions. More importantly, the sample characteristics between the two studies differed considerably. Künecke et al. [30] tested healthy, young, well-educated male and female participants, whereas this study was limited to males who were on average 10 years older and had a substantially poorer educational background. One could argue that the current sample seems to have a bias to process neutral expressions as more negative (e.g., akin to patients with Social Anxiety Disorder; [82]), thereby blurring the differences between angry or sad and neutral expressions. An additional means of analysis would have been signal detection theory (e.g., [83]). As the focus of the current paper is to test a measurement model of facial muscle responses by Künecke et al. [30] in psychopathic individuals, the signal detection theory approach is out of the scope of the present work. Future research could profit by using the method since it might be more sensitive to capture group differences.

A second possible limitation of the study is that we did not investigate facial responses to fearful expressions, which are thought to be disrupted in psychopathic individuals (e.g. [32]). The prior evidence identifying a fear-specific deficit has been challenged by more recent reviews, however, with researchers noting a deficit in the perception of fear comparable in magnitude for the perception of sadness [33, 34]. More importantly, fear was excluded because, based on previous research, recording of corrugator activity would presumably not have allowed capturing immediate facial muscle responses to the perception of fearful expressions, and there are no other facial muscles that are consistently activated when perceiving fear. Thus, measuring facial responses to fearful expressions with EMG does not seem to work reliably [47, 48], a result that is supported by studies using methods akin to the approach chosen here [30].

### Implications

The present evidence is at odds with the IES theory [5] according to which psychopathy is linked to processing deficits of distress that are associated with the amygdala. Non-offender, low and high psychopathy groups did not differ in their corrugator response to facial expressions of anger, happiness, or sadness. Our results thus support the equivalence of magnitude in responding to facial expressions–a precursor to empathy [8, 10]–among psychopaths compared to non-psychopaths. This does not exclude potential amygdala-associated processing differences in other emotion response systems like heartrate or skin conductance.

The lack of group differences suggests psychopaths will perform similarly to non-psychopaths in emotion recognition, even when task difficulty is higher than in our study and emotion simulation might become functional [13, 30]. However, the relation between immediate facial responsiveness and emotion recognition could be moderated by psychopathy. This question will be addressed in future investigations.

Although current results suggest that psychopaths’ facial muscles are as responsive as those of other people (at least towards angry, happy, neutral, and sad expressions), psychopaths may nevertheless fail to consider the emotional states of others in their own decision-making. This could either be due to a kind of egocentric override (i.e., they know but do not care) or alternatively, to an attentional deficit when they ought to process two types of information simultaneously (i.e., cues for individual goal-directed behavior and the emotional responses of others associated with this behavior; [84]). According to Newman’s response modulation hypothesis [85, 86], psychopaths pose an emotion paradox because they may appraise emotions accurately but barely use them in their decision-making. Additionally, our results do not necessarily mean that psychopaths will respond as empathically as others toward displays of anger, happiness, or sadness. Future research should strive to use images showing emotional facial expressions as distracters in decision-making tasks that include positive reinforcement. This could provide a differential test of Blair’s IES theory and Newman’s response modulation hypothesis.

## Supporting information

S1 Fig Results of structural equation modeling. (DOCX)

S1 Tables Results of confirmatry group analysis. (DOCX)
