# Supplementary material for: Facial responsiveness of psychopaths to the emotional expressions of others
Source: PLoS One. 2018 Jan 11;13(1):e0190714. doi: 10.1371/journal.pone.0190714 (PMC5764293; doi:10.1371/journal.pone.0190714)
Supplement: S1 Fig — (DOCX) [file pone.0190714.s001.docx]

Supporting information S1 Fig to “Facial responsiveness of psychopaths to the emotional expressions of others”, *PLOSONE*

Janina Künecke^12*^, Andreas Mokros^3^, Sally Olderbak^4^, and Oliver Wilhelm^4^

^1^Department of Psychology, Humboldt-Universität zu Berlin, Germany

^2^Psychologische Hochschule, Berlin, Germany

^3^University Hospital of Psychiatry Zurich, Department for Forensic Psychiatry, Zurich, Switzerland

^4^Department of Psychology, Ulm University, Ulm, Germany

*Corresponding author:

E-mail: [j.kuenecke@psychologische-hochschule.de](mailto:janina.kuenecke@hu-berlin.de) (JK)

**Results of Structural Equation Model**

In this supplementary material, we provide structural equation model (SEM) results relating psychopathy, treated as a dimensional construct, with FACE and the emotion-specific response factors ANG, HAP, and SAD of the corrugator measurement model. For the measurement of psychopathy, a bifactor model, as proposed by Patrick, Hicks, Nichol, and Krueger [1], fitted the data best [2]. In this model, the general factor PSY captures the common variance of all PCL: SV items and thus represents a continuous measure of general psychopathy, while the INT and the LIF/SOC factor subsume the specific variance of the items of the interpersonal domain (PCL: SV items 1-3) and the lifestyle/antisocial component (PCL: SV items 7-12) of psychopathy, respectively. In the SEM, depicted in S1 Fig below, we tested correlations of the general psychopathy factor with all corrugator response factors. The model fit the data well (χ^2^_(227)_ = 256.47, *p* = .09, CFI = .99, RMSEA = .02, SRMR = .04) and all factor loading were statistically significant. Construct reliabilities of all factors, measured with weighted omega Ω_W_ [3], were acceptable: Ω_W_ PCL = .90, Ω_W_ INT = .51, Ω_W_ LIFE/SOC = .69, Ω_W_ FACE = .80, Ω_W_ ANG = .59, Ω_W_ HAP = .89, and Ω_W_ SAD = .66. The correlations between PSY with FACE (*r* = -.05, *p* = .50), ANG (*r* = -.01, *p* = .96), HAP (*r* = -.07, *p* = .33), and SAD (*r* = -.07, *p* = .41) were not different from zero. The lack of a relation between psychopathy with all corrugator response factors is consistent with the group comparisons reported in the main article and speaks against a reduced facial responsiveness in psychopathic individuals to facial expressions of emotions.


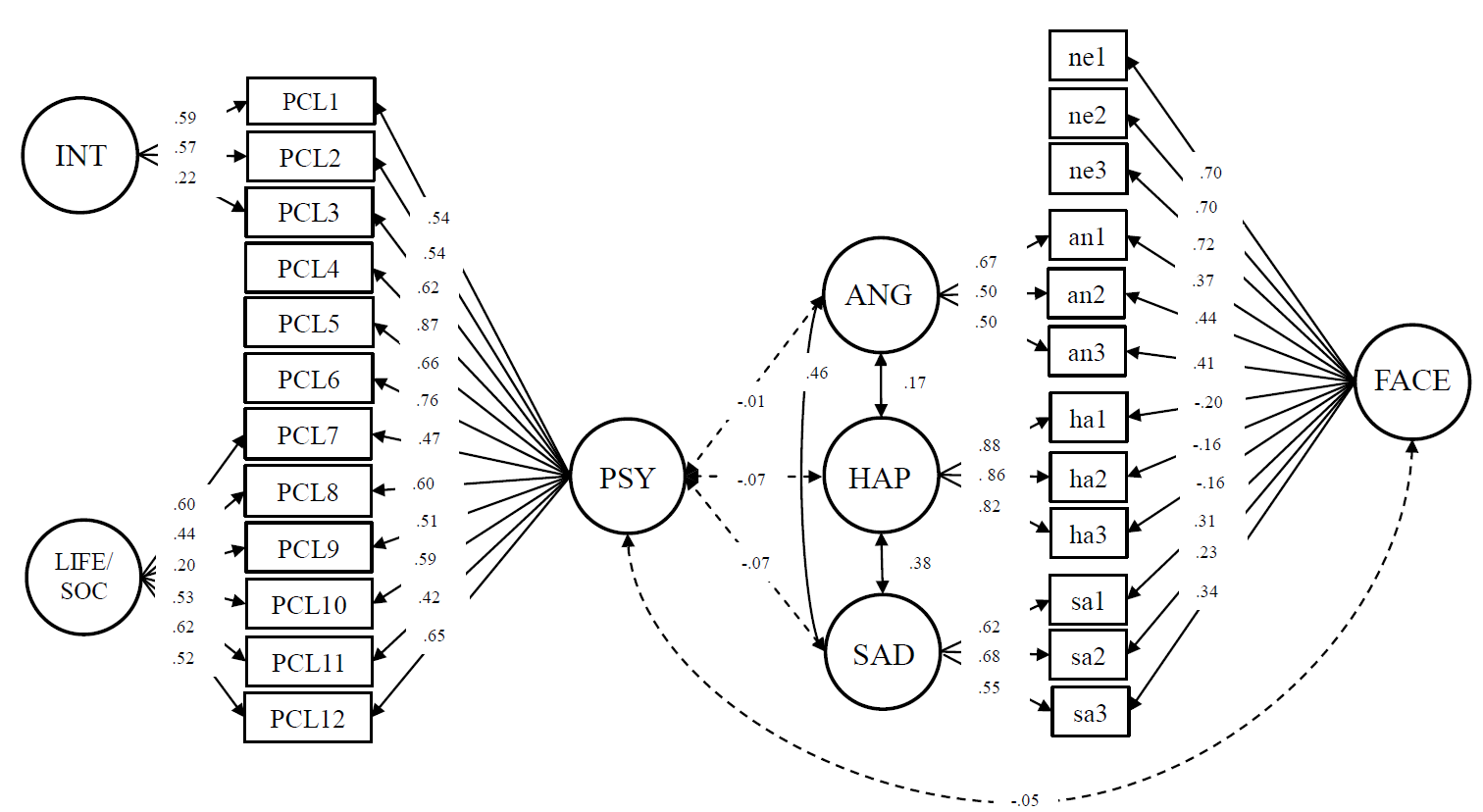


*S1 Fig.* SEM testing the correlations between general psychopathy (PSY) and corrugator response factors (FACE, ANG, HAP, and SAD). Dashed lines indicate non-significant correlations.

**References**

1. Patrick CJ, Hicks BM, Nichol PE, Krueger RF. A bifactor approach to modeling the structure of the psychopathy checklist-revised. Journal of personality disorders; 2007; 21(2), 118[: 10.1521/pedi.2007.21.2.118](https://doi.org/10.1521/pedi.2007.21.2.118)

2. Mokros A, Kovacevic A, Olderbak S, Habermeyer E, Nitschke J, Wilhelm O. Psychopathic traits in correctional and community samples: A network perspective. Manuscript in preparation. 2017.

3. McDonald RP. Test theory: A unified treatment. Mahwah NJ: Lawrence Erlbaum Associates. 1999.
