# Supplementary material for: Facial responsiveness of psychopaths to the emotional expressions of others
Source: PLoS One. 2018 Jan 11;13(1):e0190714. doi: 10.1371/journal.pone.0190714 (PMC5764293; doi:10.1371/journal.pone.0190714)
Supplement: S1 Tables — (DOCX) [file pone.0190714.s002.docx]

Supporting information S1 Tables to “Facial responsiveness of psychopaths to the emotional expressions of others”, *PLOSONE*

Janina Künecke^12*^, Andreas Mokros^3^, Sally Olderbak^4^, and Oliver Wilhelm^4^

^1^Department of Psychology, Humboldt-Universität zu Berlin, Germany

^2^Psychologische Hochschule, Berlin, Germany

^3^University Hospital of Psychiatry Zurich, Department for Forensic Psychiatry, Zurich, Switzerland

^4^Department of Psychology, Ulm University, Ulm, Germany

*Corresponding author:

E-mail: [j.kuenecke@psychologische-hochschule.de](mailto:janina.kuenecke@hu-berlin.de) (JK)

**Results of correlational group differences**

In this supplementary material, we provide explorative results on group differences in the correlations of the emotion-specific response factors. For this, in the model with equal factor means (model 4) all factor correlations were fixed to equality. This led to a significantly worse model fit (Δχ^2^_(6)_ = 20.61, *p* = .002, ΔCFI = .002), indicating the relations between the emotion-specific latent factors differed between the groups. To identify which relations were significantly different, we conducted successive model comparisons where invariance of the factor correlations was tested separately for each correlation. The model comparisons are shown in table S1. The ANG-HAP facial response factor correlation did not differ between groups (Δχ^2^_(2)_ = .12, *p* = .94, ΔCFI = -.002), while the ANG-SAD (Δχ^2^_(2)_ = 8.46, *p* = .014, ΔCFI = .005) and the HAP-SAD (Δχ^2^_(2)_ = 10.98, *p* = .004, ΔCFI = .007) correlations did. Table S2 depicts the latent factor correlations for the three groups.

To test correlation differences between the specific groups, we compared models in which the ANG-SAD or the HAP-SAD correlations were fixed for only two groups against a model where those correlations were fixed for all three groups (in all of these models, the ANG-HAP correlation was fixed between all three groups). These analysis showed that the HAP-SAD correlation was equal between the non-offender and the low psychopathy group (Δχ^2^_(1)_ = .91, *p* = .34, ΔCFI = 0), but was significantly stronger for the high psychopathy group (Δχ^2^_(1)_ = 7.55, *p* = .006, ΔCFI = .005). Additionally, the ANG-SAD correlation was equal between the non-offender and the high psychopathy group (Δχ^2^_(1)_ = .71, *p* = .40, ΔCFI = 0), but was significantly stronger for the low psychopathy group (Δχ^2^_(1)_ = 10.27, *p* = .001, ΔCFI = .001).

Since differences in correlations can be driven by differences in variances [1,2], we retested all of the model comparisons by fixing factor variances both with and without fixing the factor correlations. Fixing all variances to equality, while allowing the factor correlations between groups to be freely estimated, still led to a significantly worse model fit (Δχ^2^_(8)_ = 23.28, *p* = .003, ΔCFI = .013). Specifically, the variance of the FACE and SAD factors differed between the experimental groups with the variance for both factors higher in the high psychopathy group. Additional model comparisons regarding the correlations (with equal factor means *and* variances) showed substantial correlation differences for the ANG-SAD (Δχ^2^_(2)_ = 17.04, *p* < .001, ΔCFI = .013) and the HAP-SAD facial response factor correlations (Δχ^2^_(2)_ = 7.17, *p* = .02, ΔCFI = .005) suggesting that the significant differences between groups regarding those factor correlations were not due to differences in factor variances.

**Discussion of correlational group differences**

Although there were no factor mean differences (see manuscript), we found that the emotion-specific factor correlations differed between groups. For the high psychopathy group, the correlation between the facial responses to sad and happy expressions was higher in comparison to the non-offender and the low psychopathy group, however the correlations between sad and happy with anger were not significantly lower for the high psychopathy group. These results could be explained by research relating the amygdala to the processing of happy and sad, but not angry, facial expressions [3]. Only in the high psychopathy group were the predominantly amygdala-processed expressions (happy and sad) less related to the non-amygdala-processed expression (angry). This pattern of idiosyncratic factor correlations for individuals scoring high in psychopathy could be an indicator of their deficient physiological response to emotional stimuli, which might interfere with appropriate stimuli-reinforcement associations [4]. Clearly, this explanation requires further empirical evidence and theoretical elaboration on the interpretation of facial response factor correlations.

For the low psychopathy group, the correlation of facial responses to angry and sad expressions was quite high, while correlations were around .20 for happy and sad expressions and happy and angry expressions. Thus, offenders scoring low in psychopathy who showed strong corresponding facial responses to angry expressions also showed strong corresponding facial responses to sad but not to happy expressions. It seems like the positive-negative distinction of facial expressions was more salient for the incarcerated low psychopathy group than for the non-offender and high psychopathy group. One might speculate that for incarcerated people, congruent facial responses to negative expressions have different consequences than congruent facial responses to positive expressions - both for their inner emotional state as well as for the interaction with their counterpart.

In Künecke et al. [5] all correlations between angry, happy, and sad factors were high. In contrast, the correlations in the present study were rather low. This might be due to differences in the experimental setting (presented emotion categories) and sample characteristics (sex, age, mental health, and educational background) as outlined in the manuscript. A comparison of our results with Künecke et al. [5] endorse the idea that analyzing changes in factor correlations as a function of variables like psychopathy is an interesting approach to test theories of embodiment.

**References**

1. Little TD, Slegers DW, Card NA. A non-arbitrary method of identifying and scaling latent variables in SEM and MACs Models. Structural Equation Modeling. 2006; 13:59-72. DOI: 10.1207/s15328007sem1301_3

2. Morris SB, Methods of Meta-Analysis: Correcting Error and Bias in Research Findings. 2007; Sage Publications.

3. Fusar-Poli P, Placentino A, Carletti F, Landi P, Allen P, Surguladze S, Barale F, et al. Functional atlas of emotional faces processing: Avoxel-based meta-analysis of 105 functional magnetic resonance imaging studies. Journal of Psychiatry & Neuroscience. 2009; 34:418.

4. Blair RJ. The emergence of psychopathy: Implications for the neuropsychological approach to developmental disorders. Cognition. 2006; 101:414-442. DOI: 10.1016/j.cognition.2006.04.005

5. Künecke J, Hildebrandt A, Recio G, Sommer W, Wilhelm O. Facial EMG Responses to emotional expressions are related to emotion perception ability. PloS ONE; 2014; 9(1):e84053. DOI: 10.1371/journal.pone.0084053

| S1 Table. *Results for equality constraints on factor means and correlations.* | | | | | | | | | | | | | | | | | | | | |
| --- | --- | --- | --- | --- | --- | --- | --- | --- | --- | --- | --- | --- | --- | --- | --- | --- | --- | --- | --- | --- |
| Equality constraints | | χ^2^ | | df | | CFI | | RMSEA | | SRMR | | Compared to model | | Δχ^2^ | | Δdf | | *p*-value | Δ CFI | |
| 4) Factor means | | 200.23 | | 184 | | .986 | | .032 | | .089 | |  | |  | |  | |  |  | |
| 5) ANG-HAP correlation | | 200.35 | | 186 | | .988 | | .030 | | .089 | | 4 | | .12 | | 2 | | .94 | -.002 | |
| 6) ANG-HAP and ANG-SAD correlation | | 208.82 | | 188 | | .982 | | .036 | | .107 | | 5 | | 8.46 | | 2 | | .01 | .006 | |
| 7) ANG-HAP and HAP-SAD correlation | | 211.33 | | 188 | | .980 | | .038 | | .100 | | 5 | | 10.98 | | 2 | | .004 | .008 | |
| S2 Table. *Latent factor correlations in the model with scalar measurement invariance; Covariances, SE, correlations* | | | | | | | | | | | | | | | | | | | |  |
| Latent Factor correlation | Non-offenders | | | | | | Low psychopathy | | | | | | High Psychopathy | | | | | | |  |
|  | *cov* | | *SE* | | *r* | | *cov* | | *SE* | | *r* | | *cov* | | *SE* | | *r* | | |  |
| ANG-HAP | .005 | | .004 | | .210 | | .004 | | .004 | | .111 | | .006 | | .004 | | .225 | | |  |
| ANG-SAD | .005 | | .002 | | .451* | | .012 | | .003 | | .870* | | .002 | | .003 | | .125 | | |  |
| HAP-SAD | .008 | | .003 | | .365* | | .003 | | .004 | | .134 | | .024 | | .006 | | .601* | | |  |
| ** indicate p-values < .05* | | | | | | | | | | | | | | | | | | | |  |
